# Telomere Length Shows No Association with *BRCA1* and *BRCA2* Mutation Status

**DOI:** 10.1371/journal.pone.0086659

**Published:** 2014-01-29

**Authors:** Emma Killick, Malgorzata Tymrakiewicz, Clara Cieza-Borrella, Paula Smith, Deborah J. Thompson, Karen A. Pooley, Doug F. Easton, Elizabeth Bancroft, Elizabeth Page, Daniel Leongamornlert, The IMPACT collaborators, Zsofia Kote-Jarai, Rosalind A. Eeles

**Affiliations:** 1 Division of Medical Oncology, University Hospital Southampton NHS Foundation Trust, Southampton, United Kingdom; 2 Oncogentics, Institute of Cancer Research, Surrey, United Kingdom; 3 Unidad de Medicina Molecular, Universidad de Salamanca, Salamanca, Spain; 4 Centre for Cancer Genetic Epidemiology, University of Cambridge, Cambridge, United Kingdom; 5 Cancer Genetics, Royal Marsden Hospital NHS Trust, London, United Kingdom; Department of Medicine and Biomedical Sciences, University of Algarve, Portugal

## Abstract

This study aimed to determine whether telomere length (TL) is a marker of cancer risk or genetic status amongst two cohorts of *BRCA1* and *BRCA2* mutation carriers and controls. The first group was a prospective set of 665 male *BRCA1/2* mutation carriers and controls (mean age 53 years), all healthy at time of enrolment and blood donation, 21 of whom have developed prostate cancer whilst on study. The second group consisted of 283 female *BRCA1/2* mutation carriers and controls (mean age 48 years), half of whom had been diagnosed with breast cancer prior to enrolment. TL was quantified by qPCR from DNA extracted from peripheral blood lymphocytes. Weighted and unweighted Cox regressions and linear regression analyses were used to assess whether TL was associated with *BRCA1/2* mutation status or cancer risk. We found no evidence for association between developing cancer or being a *BRCA1* or *BRCA2* mutation carrier and telomere length. It is the first study investigating TL in a cohort of genetically predisposed males and although TL and *BRCA* status was previously studied in females our results don't support the previous finding of association between hereditary breast cancer and shorter TL.

## Introduction

Telomeres are hundreds to thousands of nucleotide repeats (TTAGGG) located at eukaryotic chromosome ends [1], without which genetic material would be lost every time a cell divides. The capacity of a cell to replicate is limited by TL; when telomeres become critically short, cell cycle arrest, senescence and apoptosis is signalled [2]. If this process fails for some reason and cell division persists despite the telomeres being too short then chromosomal instability results with end-to-end fusion of chromosomes [3].

Many factors are known to be associated with reduced TL, a primary factor being age with peripheral blood lymphocyte telomeres shortening by, on average, 41 base pairs per year [4]. Various studies have shown other factors associated with TL including smoking [5], inflammatory processes, socio-economic status, diet [6], BMI [7], diabetes mellitus [8], coronary artery disease, ulcerative colitis [9], physical activity and psychological stress [10].

Conflicting results have come from studies investigating links between cancer risk and TL. A recent meta-analysis demonstrated an association between shorter TL and increased cancer risk in studies of bladder cancer, oesophageal cancer, gastric cancer, head and neck cancer, ovarian cancer and overall incident cancers [11]. There was inconsistency in studies looking at association between TL and risk of non-Hodgkins lymphoma, breast cancer, lung cancer and colorectal cancer; the single study in prostate cancer was negative. That study looked at leukocyte TL as a marker of prostate cancer risk within the participants of the Prostate, Lung, Colorectal and Ovarian (PLCO) screening trial [5]. In the main analysis prostate cancer cases and controls did not differ with respect to TL, although there was a trend to decreased risk of prostate cancer with shorter TL once adjusted for smoking status. Interestingly, in individuals with a family history of prostate cancer shorter telomeres seemed to be associated with increased prostate cancer risk, although again this did not reach significance. Published studies investigating breast cancer risk and TL have produced conflicting results, with two studies showing a significant association between longer telomeres and breast cancer risk [12], [13] but other studies showing no association, or association only in sub-groups [14]–[16]. A potential confounding factor is whether such studies are undertaken retrospectively or prospectively, with both chemotherapy and radiotherapy having the potential to affect peripheral blood lymphocyte TL [17], [18]. A recently published prospective study of over 47000 individuals followed-up for 20 years found an association between shorter telomere length and survival once diagnosed with cancer, but no association with cancer risk [19].

Both prostate and breast cancer have a heritable component to their aetiology [20]; much of the heritability of prostate cancer is thought to be due to the inheritance of multiple low penetrance susceptibility single nucleotide polymorphisms (SNPs), but inheritance of rarer *BRCA1* or *BRCA2* gene mutations is known to increase breast cancer risk and to a lesser extent prostate cancer risk. A recent paper demonstrated reduced TL in breast cancer patients who carried mutations in the *BRCA1/2* genes compared with sporadic breast cancer cases [21]. The same group also demonstrated shorter TL in both sporadic and hereditary ovarian cancer compared with controls [22]; no similar investigation has been carried out in male *BRCA1/2* mutation carriers with prostate cancer. Preclinical studies have suggested that BRCA2 has a role in telomere stabilisation [23]–[25]. Also some studies have shown that *BRCA1* expression may have an effect on TL [26], [27], although the evidence is not as strong as for *BRCA2*. Both papers from Delgado-Martinez *et al*. included larger numbers of cases with mutations in *BRCA1* than in *BRCA2*. In this study, we have analysed TL in two distinct cohorts; a retrospective female cohort of *BRCA1/2* mutation carriers and their non-carrier relatives, a proportion of whom have a previous diagnosis of breast cancer, and a prospective male cohort of *BRCA1/2* mutation carriers and controls, all cancer free at time of blood donation, a proportion of whom have developed prostate cancer during follow-up. Although *BRCA1/2* mutations are predominantly associated with breast and ovarian cancer risk the risk of prostate cancer is increased in males, and TL may be useful for risk stratification. The association between genetic status and TL should not be affected by retrospective or prospective nature of the studies.

## Materials and Methods

### Ethics statement

IMPACT and RMH Carrier Clinic Set were approved by the National Health Service, Health Research Authority, National Research Ethics Service, London (reference numbers 05/MRE07/25 and 05/Q0801/7 respectively); all participants gave informed written consent.

### Study cohorts

#### 1. IMPACT set

TL was measured in blood DNA from male *BRCA1* and *BRCA2* mutation carriers and controls from the IMPACT study (Identification of Men with a genetic predisposition to ProstAte Cancer: Targeted Screening in *BRCA1/2* mutation carriers and controls), an international study set up to evaluate PSA screening in male *BRCA1* and *BRCA2* carriers. To be eligible, men must be either a *BRCA1* or *BRCA2* mutation carrier or from a family harbouring the gene mutation but have tested negative themselves (controls). All men were aged between 40 and 69 years with no history of prostate cancer, and no previous biopsy for raised PSA. The recruits gave a blood sample for DNA extraction at enrolment and underwent annual PSA screening with PSA>3.0 ng/ml triggering a diagnostic prostate biopsy. A total of 240 *BRCA1* and 207 *BRCA2* male mutation carriers plus 218 controls were used in a prospective study of TL. As only four controls developed prostate cancer these were removed from the analyses i.e. the association between TL and prostate cancer risk was not tested in the non-carriers. Smoking status was divided into four categories (no, ex-smoker, yes currently, not known) with ‘no’ being the baseline category in the analyses.

#### 2. Royal Marsden Hospital (RMH) Carrier Clinic set

*BRCA1/2* mutation carriers and controls (non-carrier family members) were recruited to the RMH Carrier Clinic set, in which they donate blood samples for DNA extraction at enrolment. To be eligible, recruits must be of known *BRCA1/2* mutation status and d over 18 years. Only samples from female recruits were used for the TL analysis. A total of 131 *BRCA1* and 109 *BRCA2* female mutation carriers plus 43 controls were used in a retrospective study of TL. One person was found to have mutations in both genes so was included in the analyses as a *BRCA1* mutation carrier.

### Telomere length quantification

Using quantitative real-time PCR, relative TL was measured in DNA commercially extracted from peripheral blood lymphocytes. 20 ng of DNA per sample was dried down in 384 plates. Each plate also contained serial dilutions of DNA giving 100ng DNA to 1ng DNA in duplicate to allow for efficiency calculations (efficiency = 10^(−1/slope)^) and three reference DNAs were added to each plate. All samples were tested in duplicate. The primer sequences quantifying TL were: 5′ CGGTTTGTTTGGGTTTGGGTTTGGGTTTGGGTTTGGGTT 3′ (TEL_F) and 5′ GGCTTGCCTTACCCTTACCCTTACCCTTACCCTTACCCT 3′ (TEL_R) (Integrated DNA Technologies) [28].

The single copy gene used as a control (CON) was *36B4* (gene that encodes acidic ribosomal phosphoprotein) which had the advantage that it could be amplified under the same PCR conditions as the TEL, thus reducing the risk of inter-plate variation between the TEL and CON reactions. The *36B4* primer sequences were 5′ CAGCAAGTGGGAAGGTGTAATCC 3′ (36B4_F) and 5′ CCATTCTATCATCAACGGGTACAA 3′ (36B4_R) (Integrated DNA Technologies) [29]. 5 µl of 2× power SYBR Green PCR Master Mix (KapaBiosystems) and 4 µl H_2_O were added to each well; samples were left for two hours in the dark at 4°C for resuspension of DNA. The thermal cycling profile proceeded as follows: 10 minutes at 95°C followed by 35 cycles of 95°C for 15 seconds, 54°C for 2 minutes and 72°C for 15 seconds as used previously [21]. The qPCR amplification was performed using Applied Biosystems 7900HT real-time PCR system and the RQ Manager software version 1.2 was used to determine the relative TL.

The Ct value, defined as the number of PCR cycles taken for the amplified DNA to cross a predefined threshold, was used to calculate the telomere repeat copy number to single copy gene copy number ratio (T/S) using *T/S  = 2^−ΔCt^*. The relative TL was determined by normalizing the ratio (T/S) of each sample to the calibrator DNA to standardise sample values across all reaction plates. For ease of data manipulation the natural log was taken of this value. Three internal controls DNA were run in each plate. For each run, PCR efficiency and internal controls values were monitored.

### Statistical analysis

#### Age

Linear regression analysis was used in both sets to assess the association between age and TL. Correlation was assessed using Spearman's correlation.

#### Carrier status

Linear regression analyses were used to correlate TL with carrier status. The analyses were adjusted for age at enrolment and smoking status in the IMPACT set, and adjusted for age in the RMH Carrier Clinic set (smoking status was not available for the female set); analyses were carried out using a robust variance estimation to account for several individuals coming from the same family. The joint analysis of both *BRCA1* and *BRCA2* mutation carriers was stratified by gene.

#### Cancer Risk

Hazard ratios (HR) for prostate cancer according to TL in the IMPACT study were estimated using Cox regression, adjusting for smoking status and age at blood draw. Age at enrolment was used as the left-censor age.

Women in the RMH Carrier Clinic set were recruited on the basis of having a *BRCA1/2* mutation or having a relative with such a mutation. A diagnosis of breast cancer, particularly at a young age, would very often be part of the criteria for genetic testing, and since women with breast cancer prior to recruitment were not excluded it is likely that cases of breast cancer would be overrepresented in the set. HRs from a standard Cox regression approach would be potentially biased, so we instead used a weighted cohort Cox regression analysis [30]. Individuals were weighted such that the observed breast cancer incidence rates in each age interval in the study samples were consistent with established external estimates of cancer risk among *BRCA1* and *BRCA2* mutation carriers in that age interval. As the study group was small and the majority of individuals (94%) were aged less than 60 years, weights were calculated using the post-1950 incidence rates. The analyses were carried out using a robust variance estimation to account for several individuals coming from the same family and were adjusted for age at blood draw. The joint analysis of both *BRCA1* and *BRCA2* mutation carriers was stratified by gene and the *BRCA1* mutation incidence rates were used to calculate the weights.

## Results

### IMPACT set

**Figure 1:**
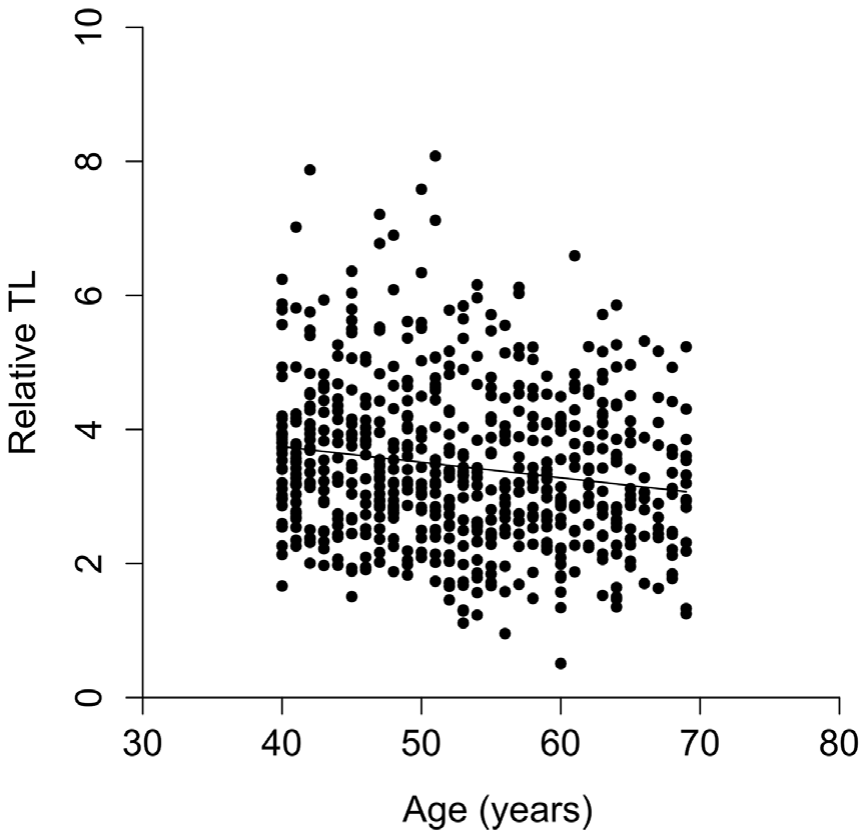
Correlation between relative TL of unaffected men in IMPACT set (n = 644) and age at blood-draw (r = −0.164, p<0.001).

Participants' characteristics are summarised in Table 1. There was an approximately equal division of men between *BRCA1* mutation carriers, *BRCA2* mutation carriers and controls; 21 men had prostate cancer. The expected negative correlation between TL in and age was observed among those unaffected by cancer (r = −0.164, p<0.001), see Figure 1.

**Table 1:** Characteristics of IMPACT and RMH Carrier Clinic recruits.

	IMPACT	RMH Carrier Clinic set
N	665	283
Mean age (range)	53.0 (40–69)	48.0 (20–78)
*BRCA1* mutation carriers	240	131
*BRCA2* mutation carriers	207	109
Controls	214	43
Affected by cancer (% of *BRCA1/2* mutation carriers affected)	21 (4.7%)	124 (51.7%)
Mean age at cancer diagnosis (range)	53.1 (41–68)	42.7 (23–69)

We investigated if there was any association between *BRCA* mutation status and TL, analysing *BRCA1* and *BRCA2* mutation carriers separately and combined, and found no significant association, see Table 2. There was >90% power to detect a difference in TL of 10%.

**Table 2:** Association between *BRCA1* or *BRCA2* mutation status and mean relative TL in the IMPACT study, adjusting for age and smoking status, and in the RMH Carrier Clinic Set, adjusting for age.

Cohort	Genetic status	n	Adjusted Mean rel TL (se)	P vs non-carriers
*IMPACT set*	BRCA1/2 Non-carrier	214	1.17 (0.024)	-
	BRCA1 Carriers	240	1.16 (0.226)	0.638
	BRCA2 Carriers	207	1.19 (0.024)	0.768
	All BRCA1&2 Carriers	447	1.17 (0.017)	0.900
*RMH Carrier Clinic Set (all samples)*	BRCA1/2 Non-carrier	43	1.69 (0.082)	-
	BRCA1 Carriers	131	1.69 (0.041)	0.969
	BRCA2 Carriers	110	1.65 (0.043)	0.653
	All BRCA1&2 Carriers	241	1.67 (0.030)	0.815
*RMH Carrier Clinic Set (unaffected only)*	BRCA1/2 Non-carrier	43	1.72 (0.079)	-
	BRCA1 Carriers	65	1.70 (0.049)	0.809
	BRCA2 Carriers	50	1.76 (0.049)	0.746
	All BRCA1&2 Carriers	115	1.72 (0.037)	0.987

Of the 240 *BRCA1* mutation carriers, 5 developed prostate cancer, and of the 207 *BRCA2* mutation carriers, 16 individuals were diagnosed. Statistical analysis showed no association between TL and prostate cancer risk. Hazard ratios (HR), 95% confidence intervals and *P*-values for the association of telomere lengths with prostate cancer risk are shown in Table 3.

**Table 3:** Hazard ratios for prostate cancer in the IMPACT study, (Cox regression analyses) and for breast cancer in the RMH study, (weighted retrospective Cox regression analyses), by *BRCA1/2* mutation status.

Cohort	Genetic status	Number with cancer	Number without cancer	HR	95% CI	*P*
*IMPACT set*	*BRCA1* mutation carriers	5	235	1.00	0.085–11.7	0.999
	*BRCA2* mutation carriers	16	191	0.493	0.103–2.36	0.376
	*All* mutation carriers	21	426	0.658	0.188–2.30	0.512
*RMH Carrier Clinic Set*	*BRCA1* mutation carriers	66	65	1.063	0.448–2.53	0.889
	*BRCA2* mutation carriers	60	50	0.467	0.209–1.04	0.063
	*All* mutation carriers	126	115	0.782	0.449–1.36	0.384

### RMH Carrier Clinic set

**Figure 2:**
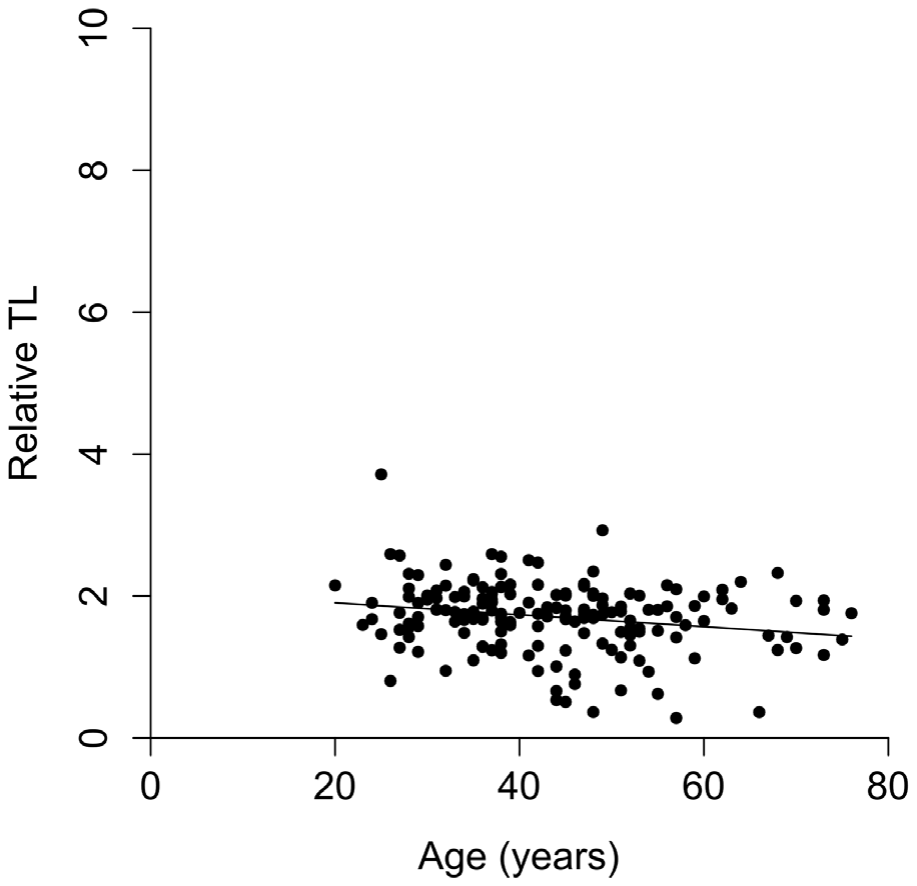
Correlation between relative TL in unaffected women in the RMH Carrier Clinic set (n = 159) and age at blood-draw (r = −0.126, p = 0.078).

The participants' characteristics of this cohort are summarised in Table 1; approximately half the subjects had been affected by cancer. There was a negative correlation between TL and age among those unaffected by cancer (r = −0.126) as shown in Figure 2, this does not quite reach significance (p = 0.078); however when a linear regression model was used to assess the impact of age on TL this was found to be significant (p = 0.001).

Stratifying by cancer status, we found no significant association between *BRCA* mutation status and TL, either considering *BRCA1* and *BRCA2* mutation carriers as separate groups, or as a combined set, see Table 2, however the power to detect a 10% difference was only 42%.

TL was compared in *BRCA1/2* mutation carriers with cancer and unaffected *BRCA1/2* mutation carriers. Statistical analysis showed no association between TL and breast cancer risk. Hazard ratios (HR), 95% confidence levels and *P*-values for the association of telomere lengths with breast cancer risk are shown in Table 3.

## Discussion

To our knowledge this is the first published study of TL in male *BRCA1/2* mutation carriers and the largest published study in female *BRCA1/2* mutation carriers. We found no association between TL and *BRCA1/2* mutation status in either of the two cohorts. Martinez-Delgado et al [21] demonstrated shorter TL in a smaller set of affected *BRCA1* mutation carriers (n = 45), *BRCA2* mutation carriers (n = 48) and healthy controls (n = 276); there was no difference between affected individuals with a family history but no mutation in *BRCA1/2* (n = 105) and the healthy control group. However, it is not clear whether the effect was due to the *BRCA1/2* mutation status or their cancer status, or indeed previous cancer treatment, as both chemotherapy and radiotherapy have been shown to affect TL [17], [18]. In order to prevent the potential confounding effect of high cancer rates we performed our analyses of the association between genetic status and TL separately for the affected and unaffected individuals. However this reduced the numbers in the analysis, increasing the chance of a false negative finding. A recently opened study is recruiting unaffected *BRCA1/2* mutation carriers to explore any relationship between TL (plus a variety of metabolic and lifestyle factors) and breast cancer risk, and will avoid the compounding factors such as treatment effects which may bias the retrospective studies [31].

In the male set it is important that the individuals underwent screening for prostate cancer, as men in the general population are not routinely screened and prostate cancer is a condition which can go undiagnosed for many years. *BRCA1/2* mutation carriers are at increased risk of developing prostate cancer and therefore, despite the limitations of screening, an unscreened cohort would be more likely to have cases of undiagnosed cancer with potential for bias.

Within the IMPACT set there was no difference in TL in *BRCA1/2* mutation carriers with prostate cancer versus cancer free *BRCA1/2* mutation carriers. This finding is in keeping with Mirabello et al [5] who did not find a difference in TL in a retrospective study of men with prostate cancer versus healthy controls. However, they did find a trend towards shorter TL in those with a family history of prostate cancer, which, in combination with the fact that TL appeared to have an effect on breast cancer risk in female *BRCA1/2* mutation carriers [21], had driven our investigation into association of TL with prostate cancer risk in male *BRCA1/2* mutation carriers. It should be noted that in this study there were only 21 cases with cancer and mean follow-up time was 46 months. Within the IMPACT study, recruits undergo biopsy based on PSA screening with a PSA>3.0 ng/ml triggering biopsy. Given the known limitations of PSA screening [32], it may be that some recruits harbour occult prostate cancer, or some are yet to develop cancer and with further follow-up there is the possibility that a difference in TL may be appreciated.

In the retrospective female *BRCA1/2* mutation carrier set, we found no significant difference in TL between those with breast cancer and cancer-free controls. There has been a discordance in previously published studies of TL in association with sporadic breast cancer, with some studies showing association and others not [14]. The only published study quantifying TL in individuals with breast cancer who carry *BRCA1/2* mutations and individuals with sporadic breast cancer showed an association between shorter telomeres in those affected by hereditary cancer, but not in those affected by sporadic breast cancer. In this study, we compared *BRCA1/2* mutation carriers with cancer and *BRCA1/2* mutation carriers without cancer, predominantly to investigate the possibility of using TL as a method of risk stratification amongst *BRCA1/2* mutation carriers. We found no evidence that TL could be useful in this way.

## Supporting Information

Supplement S1 **The IMPACT study: Identification of Men with a Genetic Predisposition to Prostate Cancer: Targeted Screening in BRCA1 and BRCA2 carriers and controls.** *The IMPACT study collaborators.*
(DOCX)
