# Supplementary material for: Telomere Length Shows No Association with BRCA1 and BRCA2 Mutation Status
Source: PLoS One. 2014 Jan 29;9(1):e86659. doi: 10.1371/journal.pone.0086659 (PMC3906069; doi:10.1371/journal.pone.0086659)
Supplement: Supplement S1 — The IMPACT study: Identification of Men with a Genetic Predisposition to Prostate Cancer: Targeted Screening in BRCA1 and BRCA2 carriers and controls. The IMPACT study collaborators. (DOCX) [file pone.0086659.s001.docx]

Supplement S1: *The IMPACT study: Identification of Men with a Genetic Predisposition to Prostate Cancer: Targeted Screening in BRCA1 and BRCA2 carriers and controls*

**Coordinating Centre, Institute of Cancer Research, London: Rosalind Eeles (PI), Elizabeth Bancroft, Elizabeth Page, Elena Castro, Natalie Taylor.**

**Recruiting sites**

**UK**

Royal Marsden NHS Foundation Trust: Susan Shanley, Audrey Ardern-Jones, Jennifer Wiggins, Kelly Kohut, Sibel Saya, Vincent Khoo, Alan Thompson, Nigel Borley.

Manchester Regional Genetics Service, Manchester: D Gareth Evans, Barbara Bulman, Jeanette Rothwell.

Wessex Clinical Genetics Service, Southampton: Diana Eccles, Catherine Mercer, Donna McBride, Philandra Costello.

East Anglian Regional Genetics Service, Cambridge: Virginia Clowes, Marc Tischkowitz, Joan Paterson, Barbara Newcombe.

Oxford Regional Genetics Service, Oxford: Lisa Walker, Dorothy Halliday, Barbara Stayner

South West Thames Regional Genetics Service, London: Shirley Hodgson, Sheila Goff, Glen Brice, Tessa Homfray.

Peninsula Clinical Genetics Service. Exeter: Carole Brewer, Alison Potter, Caroline Renton, Anne Searle, Selina Goodman.

Northern Clinical Genetics Service, Newcastle: Fiona Douglas, Irene Jobson.

South West Regional Genetics Service, Bristol: Alan Donaldson.

South East Thames Regional Genetics Service, Guys Hospital London: Louise Izatt, Gabriella Pichert, Chris Jacobs, Caroline Langman.

North West Thames Regional Genetics Service. Harrow: Angela Brady, Huw Dorkins, Athalie Melville, Carole Cummings.

North East Thames Regional Genetics Service, NE Thames: Lucy Side, Alison Male, Kate Simon.

North Trent Clinical Genetics Service, Sheffield: Jackie Cook, Louise Nevitt, Stuart Ingram

Academic Urology Unit, Sheffield: Derek Rosario, James Catto, Joanne Howson.

West Midlands Regional Clinical Genetics Service, Birmingham: Professor Cyril Chapman, Dr Trevor Cole, Tricia Heaton, Jonathan Hoffman, Lucy Burgess

West of Scotland Genetics Service, Glasgow: Dr Rosemarie Davidson, Mark Longmuir, Cathy Watt, Alexis Duncan

Leicester: Dr Julian Barwell, Kas Siguake, Beckie Speak, Zahirah Sidat

North Cumbria University Hospitals Trust: Dr Alex Henderson, Angela Birt, Una Poultney

Royal Liverpool Children’s Hospital, Liverpool: Dr Lynn Greenhalgh, Gillian Roberts

Royal Liverpool and Broadgreen Hospital NHS Trust, Liverpool : Mr Philip Cornford, Katy Treherne

**Australia**

Peter MacCallum Cancer Institute, Melbourne: Gillian Mitchell, Rebecca Doherty, Kate Drew, Jo McKinley, Lara Petelin, Sarah Pratt, Mary-Anne Young

The Walter & Eliza Hall Institute of Medical Research, Melbourne: Geoffrey Lindeman, Kylie Shackleton

Adelaide Repatriation General Hospital, Adelaide: Alan Stapleton, Jimmy Lam, Louise Taylor.

Women's and Children's Hospital, Adelaide: Graeme Suthers, Meryl Altree.

Prince of Wales Hospital, Sydney: Kathy Tucker, Robyn Ward, Lesley Andrews

Westmead Hospital, Sydney: Judy Kirk

King Edward Memorial Hospital, Perth: Sharron Townshend, Nicholas Pachter

Royal Brisbane & Women’s Hospital, Brisbane: Julie McGaughran, Rachel Susman

Royal Hobart Hospital, Tasmania: David Amor

Hunter Genetics, Newcastle, New South Wales: Allan Spigelman, Rodney Scott

Monash Medical Centre, Melbourne: Marion Harris, Mark Frydenberg

**USA**

NorthShore University HealthSystem, Evanston: Wendy Rubinstein, Tina Selkirk, Daniel Shevrin, Karen Kaul, Charles Brendler, Scott Weissman, Anna Newlin, Kristen Vogel, Suzanne O'Neill

Salt Lake City, Utah: Saundra Buys, David Goldgar, Tom Conner, Vickie Venne, Robert Stephenson, Christopher Dechet

University of Pennsylvania, Philadelphia: Susan Domchek, Jacquelyn Powers

MD Anderson, Texas: Sara Strom, Banu Arun, John W. Davis, Yuko Yamamura

Fox Chase Cancer Center: Veda Giri, Laura Gross

**Poland**

International Hereditary Cancer Centre, Szczecin: Cezary Cybulski, Jan Lubinski, Dominika Wokolorczyk

**Norway**

Norwegian Radium Hospital, Oslo: Lovise Maehle, Pal Moller, Bjorn Brennhovd, Heidi Medvik, Eldbjørg Hanslien,

**Denmark**

Vejle Hospital, Vejle: Anne-Bine Skytte,

Fredericia and Kolding Hospital, Fredericia: Palle Osther

Odense University Hospital, Odense, Anne-Marie Gerdes

**Spain**

Catalonian Institute of Oncology, Barcelona: Ignacio Blanco, Merce Peris, Mónica Salinas

Hospital de Sant Pau, Barcelona: Teresa Ramón y Cajal, David Fisas

Hospital Vall d'Hebron, Barcelona: Judith Balmaña, Neus Gadea, Juan Morote

**Malaysia**

Cancer Research Initiatives Foundation, Subang Jaya Medical Centre, Selangor Darul Ehsan: Soo Hwang Teo, Hui Meng Tan, Sook-Yee Yoon

University of Malaya, Kuala Lumpur: Soo Hwang Teo, Meow Keong Thong

**Israel**

Chaim Shema Medical Center, Tel-Hashomer: Eitan Friedman

**Italy**

Istituto Nazionale dei Tumori, Milan: Nicola Nicolai, Paolo Radice, Riccardo Valdagni, Magnani Tiziana

**Canada**

McGill University, Montreal: Marc Tischkowitz, William Foulkes, Nassim Taherian, Armen Aprikian.

**Slovakia**

National Cancer Institute, Bratislava: Lucia Copakova.

**Slovenia**

Institute of Oncology, Ljubljana: Janez Zgajnar, Mateja Krajc.

**Sweden**

Karolinska Institute, Stockholm: Annelie Liljegren, Marie Hjälm –Eriksson, Karl-Johan Ekdahl.

**India**

Tata Memorial Cancer Centrer, Mumbai: Vedang Murthy, Rajiv Sarin, Kadturi Awatagiri

**Iceland**

Landspitali, Reykjavik: Jorunn Eyfjord, Oskar Johannsson, Vigdis Stefansdottir

**The Netherlands**

STOET: Stichting Opsporing Erfelijke Tumoren, Leiden

Leiden University Medical Centre, Leiden: Christi van Asperen.

Radboud University Nijmegen Medical Centre: Bart Kiemeney.

**Portugal**

Portuguese Oncology Institute, Porto: Dr Manuel Teixeira, Sofia Maia

**Collaborating sites**

**France**

Centre Jean Perrin, Clermont-Ferrand: Yves-Jean Bignon

**Latvia**

Hereditary Cancer Institute, Riga: Andris Abele, Janis Gardovskis, Arvids Irmejs.

**Cyprus**

The Cyprus Institute of Neurology & Genetics: Kyriacos Kyriacou, Andreas Hadjisavvas.

**Turkey**

Akdeniz University, Antalya: Guven Luleci, Esra Manguoglu.

**Germany**

Universitäts-Frauenklinik zu Köln, Köln: Rita Schmutzler, Kerstin Luedtke-Heckenkamp.

**Ireland**

Mater Private Hospital, Dublin: David Gallagher, Richard Bambury, Michael Farrell, Fergal Gallagher

**USA**

Memorial Sloan Kettering Cancer Center, New York: Kenneth Offit, James Eastham, Robert Hamilton
